# Effects of 10 keV Electron Irradiation on the Performance Degradation of SiC Schottky Diode Radiation Detectors

**DOI:** 10.3390/mi15111331

**Published:** 2024-10-30

**Authors:** Jinlu Ruan, Liang Chen, Leidang Zhou, Xue Du, Fangbao Wang, Yapeng Zhang, Penghui Zhao, Xiaoping Ouyang

**Affiliations:** 1 National Key Laboratory of Intense Pulsed Radiation Simulation and Effect, Northwest Institute of Nuclear Technology, Xi’an 710024, Chinachenl_nint@163.com (L.C.);; 2 School of Microelectronics, Xi’an Jiaotong University, Xi’an 710049, China; 3 Department of Engineering Physics, Tsinghua University, Beijing 100084, China

**Keywords:** electron radiation, sillicon carbined, Schottky barrier diode

## Abstract

The silicon carbide (SiC) Schottky diode (SBD) detector in a SiC hybrid photomultiplier tube (HPMT) generates signals by receiving photocathode electrons with an energy of 10 keV. So, the performance of the SiC SBD under electron irradiation with an energy of 10 keV has an important significance for the application of the SiC-HPMT. However, studies on 10 keV radiation effects on the SiC SBDs were rarely reported. In this paper, the performance degradation of the SiC SBDs irradiated by 10 keV electrons at different fluences was investigated. After the irradiation, the forward current of the SiC SBDs increased, and the turn-on voltage decreased with the irradiation fluences until 1.6 × 10^16^ cm^−2^. According to the capacitance–voltage (*C-V*) curves, the effective doping concentration increased slightly after the irradiation, and an obvious discrepancy of *C-V* curves occurred below 5 V. Moreover, as a radiation detector, the peak position of the α-particles’ amplitude spectrum changed slightly, and the energy resolution was also slightly reduced after the irradiation due to the high collection charge efficiency (CCE) still being larger than 99.5%. In addition, the time response of the SiC SBD to the 50 ns pulsed X-ray was almost not affected by the irradiation. The results indicated that the performance degradation of the SiC SBD irradiated at the fluence of 1.5 × 10^17^ cm^−2^ would not result in a deterioration of the properties of the SiC-HPMT and showed an important significance for the supplement of the radiation resistance of the SiC SBD radiation detector.

## 1. Introduction

The hybrid photomultiplier tube (HPMT) and the hybrid photon detector (HPD) have the most striking advantages in that they have much lower statistical spread in the amplitude of the pulse outputs than conventional photomultiplier tubes [1]. One significant advantage of the superior statistical behavior is its ability to better separate a single-photoelectron-caused event from the events arising from multiple photoelectrons. So, the HPMT is often applied when the detected light levels are very low [2,3,4,5]. The HPMT anode detector is usually made of silicon (Si) material. Because of the poor radiation resistance of the Si-based detector, the HPMT based on the Si-based detector was rarely applied in the measurement of the pulsed radiation field. Compared with Si-based detectors, silicon carbide (SiC)-based detectors possess a stronger radiation resistance and have been widely applied in harsh environments [6,7,8,9,10]. Liu et al. found that the 4H-SiC Schottky diode detectors that were irradiated survived the intense neutron radiation at a fluence of 1 × 10^14^ n/cm^2^ and were still effective and could be used in radiation detection [6]. In contrast, the silicon detector is hard to operate above a neutron fluence of 1 × 10^14^ n/cm^2^ and the degradation of its CCE is worse than the SiC detector after fast-neutron irradiation [11,12]. Therefore, Chen et al. have developed an HPMT based on a SiC Schottky diode (SBD) (SiC-HPMT) by taking advantage of the excellent radiation resistance of SiC-based detectors [13,14].

Research on performance degradations of SiC detectors has been conducted under different kinds of particle irradiations, such as neutrons, protons, electrons, and so on [6,10,15,16,17,18]. Although there are some divergences in the energy levels of the generated defects [19,20], the majority believe that defects are related to Si and C vacancies. According to the elastic collision, the electron energy needs to be larger than 100 keV [21,22] to make the Si and C atoms deviate from their positions in the lattices when performance degradations of SiC detectors under electron irradiation are researched [23,24,25]. So, few research studies of electrons with an energy of lower than 100 keV were reported. However, the SiC SBD detector is irradiated by the accelerated electrons whose energy is about 10 keV in the SiC-HPMT. Research on performance degradations of the SiC diode irradiated by such low energy electrons is indispensable and has an important significance for the application of the SiC-HPMT and the supplement of the radiation resistance of the SiC SBD [26]. The SiC-HPMT is applied in detecting single particles because of its superior statistical behavior and in measuring pulsed radiation fields because of its radiation resistance. The collection charge efficiency (CCE) and the energy resolution of the SiC SBD have an impact on the application of the SiC-HPMT for detecting single particles, while the response of the SiC SBD to pulsed radiation will affect the applications of the SiC-HPMT for pulsed radiation field measurements. Therefore, the performance degradations such as the CCE, energy resolution, and pulse response of the SiC SBD under different irradiation fluences of electrons with an average energy of 10 keV were studied and depicted in this paper.

## 2. Experiments

### 2.1. SiC Schottky Diodes (SBDs)

**Figure 1:**
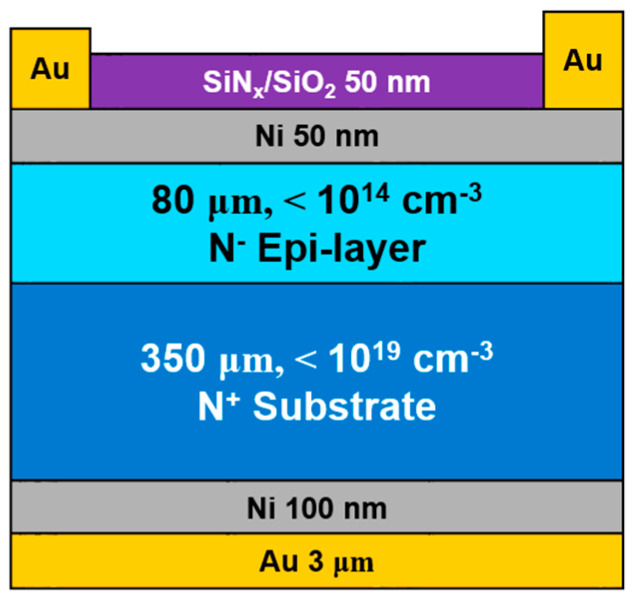
Schematic of the SiC Schottky diode.

In order to meet the requirements of the performances of the SiC-HPMT, such as fast temporal response, large linear current, low noise, and high energy resolution, the SiC detector needs to have characteristics of a fast temporal response, large linear current, low dark current, and thin dead layer. Considering the fabrication process, the choice of the SiC detector with the SBD structure is ultimately determined. As shown in Figure 1, the SiC detectors prepared by the Nanjing Electronic Devices Institute, China, for the irradiation experiments had an SBD structure, whose sensitive region was a lightly N-doped (donor doping concentration: <10^14^ cm^−3^) epitaxial 4H-SiC layer that was grown on a commercial 4H-SiC N^+^ highly conductive substrate wafer (donor doping concentration: 1 × 10^19^ cm^−3^) by chemical vapor deposition (CVD). The donor doping concentration of the epitaxial 4H-SiC needs to be as low as possible to obtain a low dark current. The concentration of 10^14^ cm^−3^ is the lowest level that the Nanjing Electronic Devices Institute can obtain by using the fabrication process at present. The SiC SBD had a diameter of 5 mm and a high-quality epitaxial layer with a thickness of 80 μm for achieving a fast temporal response of the SiC-HPMT [14]. Although J Park et al. found that the incident electrode fabricated by the Ti/Au structure showed excellent radiation tolerance [27] and other researchers found that other structures have certain advantages and disadvantages in current–voltage characteristics, production technology, and so on [28,29,30], the incident electrode was still chosen to be a structure with 50 nm thick Ni covered by multiple layers of silicon dioxide (SiO_2_)/silicon nitride (SiN_x_) (50 nm/50 nm) and Au bonding pads with a 3 μm thickness according to the requirement of the fabrication process. The multiple layers of silicon dioxide (SiO_2_)/silicon nitride (SiN_x_) (50 nm/50 nm) were used to improve the breakdown voltage, reduce the dark current, and prevent oxidation of the SiC SBD. The bottom ohmic electrode comprised a 100 nm thick Ni layer and a 3 μm thick Au layer.

### 2.2. Experiments and Measurements

The SiC SBDs were irradiated by electrons derived from the accelerator with an average energy of 10 keV at the Institute of Modern Physics, Chinese Academy of Science, Lanzhou. Three SiC detectors were exposed to different irradiation fluences, as shown in Table 1. The irradiation fluences of SiC detectors were measured by two Faraday cups with a high and a low sensitivity, respectively.

Three SiC detectors (D1, D2, and D3) from the same batch were used in the irradiation experiment to ensure that the performance of each detector was almost consistent. Electrical characteristics (such as current–voltage (*I-V*) and capacitance–voltage (*C-V*)) and radiation responses to α-particles and a pulsed X-ray source of the three SiC detectors before and after the electron irradiation were measured and compared. The D3 SiC detector was exposed to irradiation fluences varying from 10^14^ to 10^17^ cm^−2^ and its forward *I-V* curves were measured online at different irradiation fluences for obtaining its change discipline. The D1 and D2 detectors were irradiated at 10^15^ cm^−2^ and 10^16^ cm^−2^, respectively.

The *I-V* measurement used a Keysight B2902A source meter. During the measurement, the SiC detectors were placed in the vacuum chamber of the electron accelerator before and after the electron irradiation to keep the same measurement conductions. The temperature of the SiC detector was not controlled. The accelerator chamber was a light-tight chamber to prevent the influences of light. The *C-V* curves of the three SiC detectors were measured by an Agilent B1505A power device analyzer with the same parameters before and after electron irradiation.

**Figure 2:**
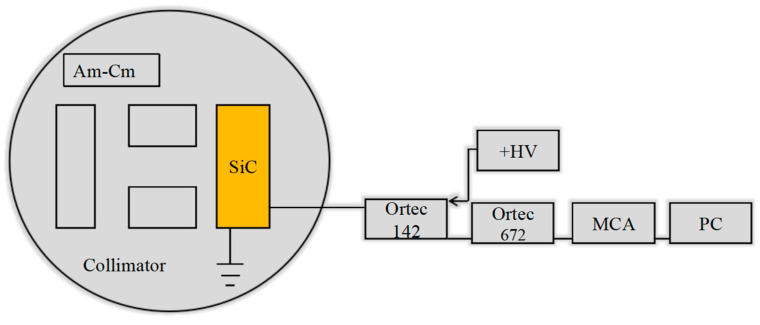
Layout of the α-particle spectra measurement system.

Figure 2 shows the α-particle spectra measurement system. The α-particle source (^243^Am-^244^Cm), the collimator with a hole of a 2 mm diameter, and the SiC detector were placed in a vacuum chamber. Alpha particles entered through the collimator entranced into the SiC detector and produced a signal amplified by the ORTEC-142 preamplifier and the ORTEC-672 amplifier. The amount of energy straggling caused by the 50 nm thick Ni layer and multiple layers of silicon dioxide (SiO_2_)/silicon nitride (SiN_x_) (50 nm/50 nm) is about 5.2 keV. The output of the ORTEC-672 amplifier was sent into the ORTEC ASPEC-927 multichannel analyzer (MCA) for recording and analyzing. The result was displayed on the PC with the MASETRO software. During the measurement, the magnification of the amplifier was 200 times, and the shaping time was 1 μs.

**Figure 3:**
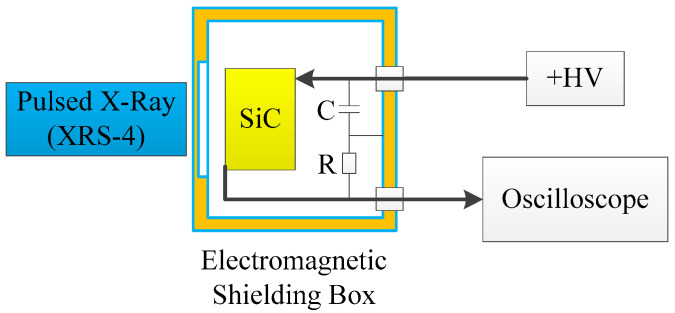
Schematic of the measurement system of responses to the pulsed X-ray source.

The responses of the SiC detector to the pulsed X-ray before and after the electron irradiation were measured by the experimental layout shown in Figure 3. The SiC detector was placed in a copper box for electromagnetic shielding. Pulsed X-rays through the copper box entered into the SiC detector to generate a signal, which was recorded by an oscilloscope (HDO8108 produced by Teledyne LeCroy) with a 1 GHz bandwidth and a 12-bit resolution.

## 3. Results and Discussion

### 3.1. I-V Characteristics

**Figure 4:**
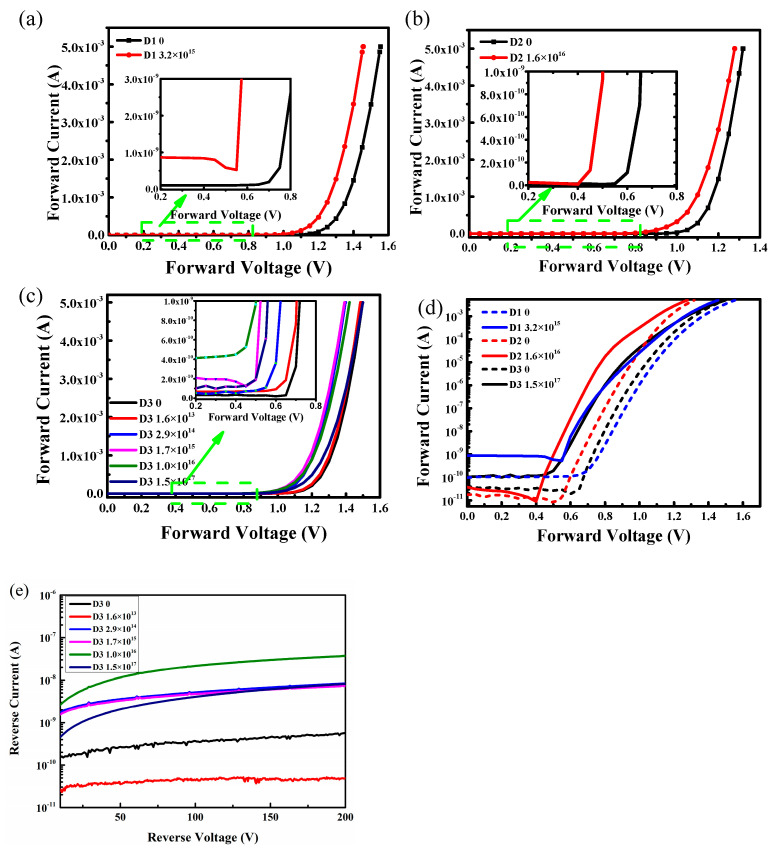
(**a**) D1 at irradiation fluences of 3.2 × 10^15^ cm^−2^. (**b**) D2 at irradiation fluences of 1.6 × 10^16^ cm^−2^. (**c**) D3 at different irradiation fluences. (**d**) Forward *I-V* characteristics of D1, D2, and D3 before and after irradiation. (**e**) Reverse *I-V* characteristics of D3 at different irradiation fluences.

The *I-V* characteristics of the three SiC SBD detectors at different irradiation fluences are shown in Figure 4. These results indicated that electron irradiation led to an increase in the forward current. According to the results, the *I-V* curves at different irradiation fluences displayed different turn-on voltages and rectification properties even at a high irradiation fluence of 1.7 × 10^17^ cm^−2^. According to the result of the D3 detector, the turn-on voltage decreased with the irradiation fluences when the irradiation fluences were smaller than 1.6 × 10^16^ cm^−2^. When the irradiation fluences reached 1.6 × 10^16^ cm^−2^, the turn-on voltage increased. The turn-on voltages of the D2 detector and the D1 detector after irradiation were 0.4 V and 0.55 V, almost the same as those of the D3 detector (0.4 V and 0.45 V) at the irradiation fluences in the same order of magnitude. The changes in forward *I-V* characteristics of the D2 and D1 detectors proved the trend in the variation of the turn-on voltage with the irradiation fluences.

The SiC SBD works under reverse bias. So, the variation of the reverse current of the SiC SBD after the electron irradiation will directly affect the performance of the SiC SBD. Figure 4e shows the reverse *I-V* characteristics of the D3 detector obtained at different irradiation fluences. When the irradiation fluence is larger than 1.6 × 10^13^ cm^−2^, the reverse current begins to increase and becomes the largest at the irradiation fluence of 1.0 × 10^16^ cm^−2^. The reverse current at the irradiation fluence of 1.5 × 10^17^ cm^−2^ is smaller than that at the irradiation fluence of 1.0 × 10^16^ cm^−2^ but still remains larger than that before the irradiation.

The defects, which may be produced by the electron irradiation or the heating during the electron irradiation [31], are responsible for the variation of the *I-V* characteristics. The reason for producing the defects is still unclear at present. In the future, we will try to confirm the reason by investigating the changes in low-temperature PL spectra of SiC materials before and after electron irradiation and high-temperature annealing, using deep-level transient spectra and other methods.

### 3.2. C-V Curves

**Figure 5:**
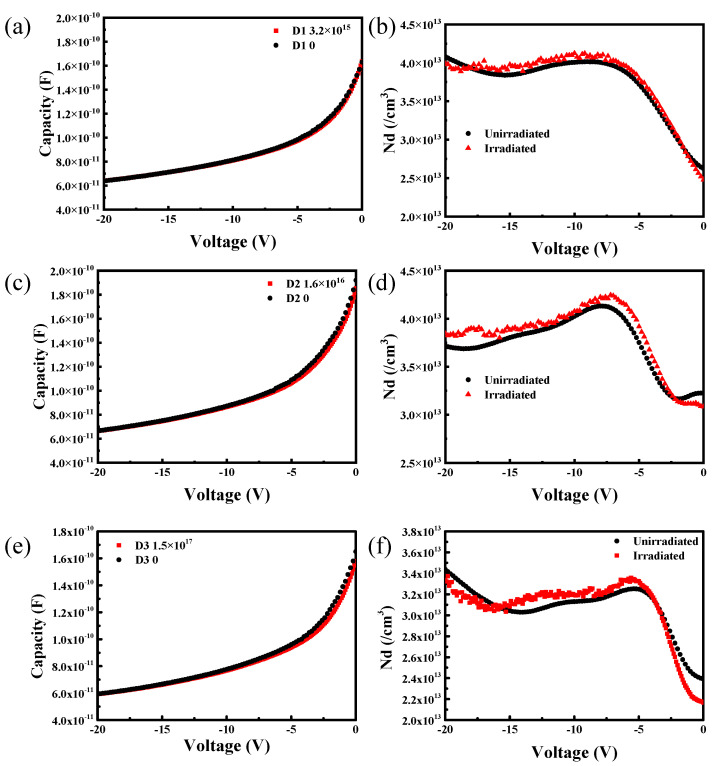
*C-V* curves and effective dopant concentrations of the three detectors before and after irradiations: (**a**,**b**) D1 detector after the irradiation at 3.2 × 10^15^ cm^−2^ irradiation fluences; (**c**,**d**) D2 detector after the irradiation at 1.6 × 10^16^ cm^−2^ irradiation fluences; (**e**,**f**) D3 detector after the irradiation at 1.5 × 10^17^ cm^−2^ irradiation fluences.

Figure 5 shows the *C-V* curves of the three detectors before and after the electron irradiation. With an increase in the irradiation fluences, the difference in the capacitances before and after the irradiation were more obvious below 5 V, while the capacitance did not change when the absolute voltage was larger than 5 V. The effective dopant concentrations (*N*_eff_) (as shown in Table 2) of the three detectors before and after radiations were calculated based on the linear segments of the 1/*C*^2^-*V* curves. The *N*_eff_ showed a slight increase after the irradiation, which may be produced by the electron irradiation or the heating during the electron irradiation [22]. Because the sensitive region was a lightly N-doped epitaxial 4H-SiC layer, more electrons would exist in the sensitive region under electron irradiations, increasing the *N*_eff_ of the detectors.

### 3.3. α-Particle Response

**Figure 6:**
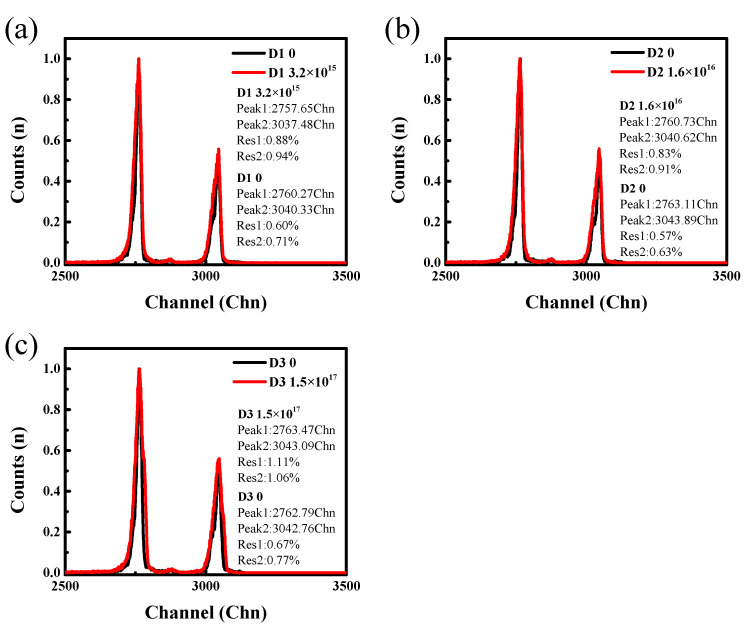
α-particle amplitude spectrum of the three detectors before and after irradiation at different irradiation fluences: (**a**) 3.2 × 10^15^ cm^−2^ irradiation fluences; (**b**) 1.6 × 10^16^ cm^−2^ irradiation fluences; (**c**) 1.5 × 10^17^ cm^−2^ irradiation fluences.

The response of the SiC detector to the single particle after the electron irradiation played an important role in the properties of the SiC-HPMT applied for detecting the single particle. So, the responses of the SiC detector to α-particles before and after the irradiation were compared. The amplitude spectra detected by the three detectors before and after the irradiation are shown in Figure 6. By comparing the spectra obtained before and after the irradiation, the energy resolution decreased slightly by about 1%, which was not significantly different from that obtained before the irradiation. When the irradiation fluences were less than 1.5 × 10^17^ cm^−2^, the peak position of the amplitude spectrum decreased by about three channels which corresponded to an equivalent energy of about 6 keV, while the peak position did not change after the irradiation generated irradiation fluences of 1.5 × 10^17^ cm^−2^.

**Figure 7:**
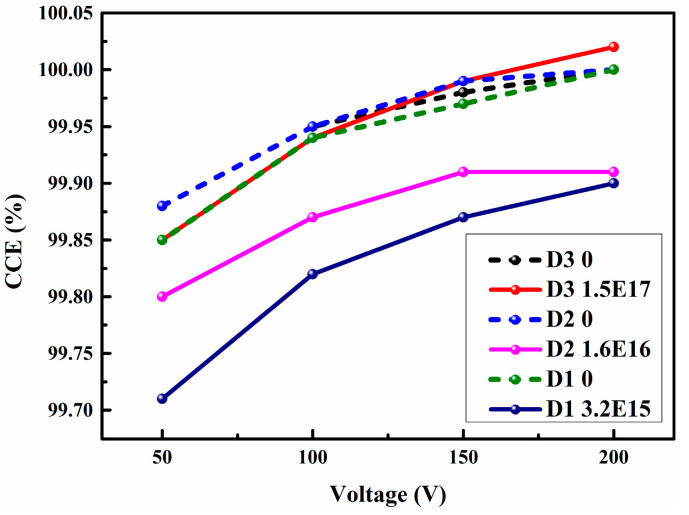
CCEs of the three detectors before and after irradiations.

The charge collection efficiency (CCE) changes before and after irradiations are shown in Figure 7, where the CCE was thought to be 100% at the bias voltage of 200 V. Although the CCE decreased after the irradiation, the CCE was still larger than 99.5%, which was the reason why the energy resolution and the peak position changed slightly after the irradiation. The maximum and minimum reductions in the CCE occurred at the irradiation fluences of 3.2 × 10^15^ cm^−2^ and 1.5 × 10^17^ cm^−2^, respectively.

### 3.4. Responses to the Pulsed X-Ray Source

**Figure 8:**
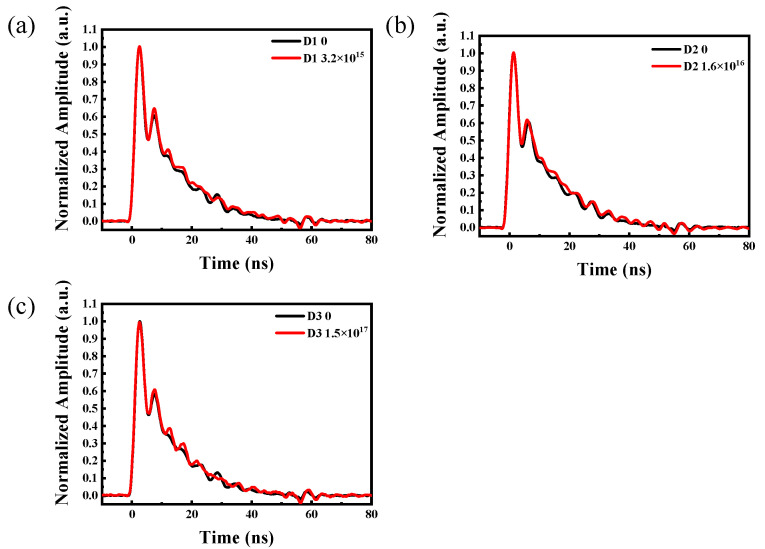
Responses of the three detectors to the pulsed X-ray source at 200V: (**a**) D1 detector; (**b**) D2 detector; (**c**) D3 detector.

The pulsed response of the SiC SBD detector, as an important effect on the properties of the SiC-HPMT, was studied by a pulsed X-ray source after the irradiation and was compared with that before the irradiation. The comparison results shown in Figure 8 indicated that the response was not affected by the electron irradiation. The waveform obtained by the D3 detector after the irradiation at a fluence of 1.5 × 10^17^ cm^−2^ still well reflected the multiple peaks characteristics of the pulsed X-ray source. The results meant that the response of the SiC-HPMT would not be affected when the SiC SBD detector was irradiated at the same fluences.

## 4. Conclusions

The SiC SBD, as a key component of the SiC-HPMT, was irradiated by the photocathode electrons, which obtained an energy of about 10 keV from the accelerating and focusing electrical field. The performance of the SiC SBD detector played a crucial role in the properties of the SiC-HPMT. So, the research on the performance degradation of the SiC SBD detector under electron irradiation with 10 keV was indispensable. These results had great significance for the application stability of the SiC-HPMT and also made a supplement of the radiation resistance of the SiC SBD.

The performance degradations of the SiC SBDs irradiated by 10 keV electrons at different fluences were investigated in this paper. Although the forward current of the SiC diode increased after the irradiation, the *I-V* curves still exhibited the rectification characteristic of the diode. The turn-on voltage of the SBD decreased with the irradiation fluences until 1.6 × 10^16^ cm^−2^ and then began to increase. The differences in *C-V* curves obtained before and after irradiations were obvious when the absolute value of the voltage was less than 5 V. The effective doping concentration increased slightly after the irradiation. After the irradiation, the peak position of the amplitude spectrum of the α-particle changed slightly (about three channels and an equivalent energy of about 6 keV), and the energy resolution was still very high, just with a little reduction of about 1%. Although the relative CCE reduced after the irradiation, it was still larger than 99.5%, so the energy resolution and the peak position changed slightly after the irradiation. The effect of the irradiation on the response of the SiC SBD detector to the pulsed X-ray source can be ignored.

In summary, the performance degradation of the SiC diode irradiated at the fluences of 1.5 × 10^17^ cm^−2^ did not result in an obvious deterioration of the properties of the SiC-HPMT. These results will be an important supplement to the radiation resistance of the SiC SBD.

## Figures and Tables

**Table 1:** Irradiation fluences of different SiC detectors.

Number of SiC Detector	D1	D2	D3
Electron fluences (cm^−2^)	3.2 × 10^15^	1.6 × 10^16^	1.5 × 10^17^

**Table 2:** The effective dopant concentrations (*N*_eff_) of different SiC detectors.

	D1 N_eff_/cm^−3^	D2 N_eff_/cm^−3^	D3 N_eff_/cm^−3^
Before	4.05 × 10^13^	4.05 × 10^13^	3.22 × 10^13^
After	4.14 × 10^13^	4.12 × 10^13^	3.27 × 10^13^

## Data Availability

The original contributions presented in the study are included in the article, further inquiries can be directed to the corresponding author.
